# The proportion of youths’ physical inactivity attributable to neighbourhood built environment features

**DOI:** 10.1186/1476-072X-12-31

**Published:** 2013-06-18

**Authors:** Rachel E Laxer, Ian Janssen

**Affiliations:** 1 School of Kinesiology and Health Studies, Queen’s University, Kingston, ON K7L 3N6, Canada

## Abstract

**Objective:**

We investigated the independent association between several neighbourhood built environment features and physical inactivity within a national sample of Canadian youth, and estimated the proportion of inactivity within the population that was attributable to these built environment features.

**Methods:**

This was a cross-sectional study of 6626 youth aged 11–15 years from 272 schools across Canada. Participants resided within 1 km of their school. Walkability, outdoor play areas (parks, wooded areas, yards at home, cul-de-sacs on roads), recreation facilities, and aesthetics were measured objectively within each school neighbourhood using geographic information systems. Physical inactivity (<5 days/week of 60 minutes of moderate-to-vigorous physical activity) was assessed by questionnaire. Multilevel logistic regression analyses, which controlled for several covariates, examined relationships between built environment features and physical inactivity.

**Results:**

The final regression model indicated that, by comparison to youth living in the least walkable neighbourhoods, the risks for physical inactivity were 28-44% higher for youth living in neighbourhoods in the remaining three walkability quartiles. By comparison to youth living in neighbourhoods with the highest density of cul-de-sacs, risks for physical inactivity were 28-32% higher for youth living in neighbourhoods in the lowest two quartiles. By comparison to youth living in neighbourhoods with the least amount of park space, risks for physical inactivity were 28-37% higher for youth living in the neighbourhoods with a moderate to high (quartiles 2 and 3) park space. Population attributable risk estimates suggested that 23% of physical inactivity within the population was attributable to living in walkable neighbourhoods, 16% was attributable to living in neighbourhoods with a low density of cul-de-sacs, and 15% was attributable to living in neighbourhoods with a moderate to high amount of park space.

**Conclusions:**

Of the neighbourhood built environment exposure variables measured in this study, the three that were the most highly associated with inactivity were walkability, the density of cul-de-sacs, and park space. The association between some of these features and youths’ activity levels were in the opposite direction to what has previously been reported in adults and younger children.

## Introduction

Over 90% of American and Canadian youth do not meet the public health guideline of 60 minutes of daily moderate-to-vigorous physical activity [1,2]. This is a problem as youths’ physical activity levels track over time [2,3] and because inactivity is related to obesity, cardiovascular and metabolic disease risk factors, bone density, and mental health outcomes [4]. In order to develop effective public health policies and interventions aimed at increasing the physical activity levels of young people, the key determinants of this behaviour need to be understood [5]. While characteristics of young people (e.g., age, gender) and their families (e.g., parental role modeling, socioeconomic status) are important determinants [6,7], so too are aspects of the neighbourhood environments in which they live [8,9].

Several features of the neighbourhood built environment are potentially relevant for physical activity. Features that facilitate walking and bicycling, such as well-connected street networks [10], mixed land use [11,12], low speed limits on roads [13], and the presence of sidewalks along roads [8] may contribute to increased active transportation but may have adverse effects on active play. Active play within young people can also be influenced by the availability of outdoor play spaces such as parks and other public green spaces and wooded areas [14-16], yards at home [17,18], and cul-de-sacs on roads [18,19]. Recreation facilities where youth can engage in organized sports may also be important determinants of physical activity levels, including community centres [20-22], arenas [22], pools [23,24], and courts/tracks [23]. Finally, aesthetic features may influence whether people want to be outdoors and engage in physical activity in their neighbourhood [25,26]. As existing studies have not simultaneously considered the impact of all of these built environment features, it is difficult to determine which features are the most strongly related to the physical activity levels of young people.

It is important to determine the relative importance of each built environment feature. This information can help direct priorities as it is not feasible to simultaneously address and improve all built environment features. From a public health perspective, the relative importance of the different built environment features can be determined by comparing their population attributable risks (PAR%). The PAR% is an estimate of the proportion of an outcome within the population, such as physical inactivity, that is attributable to a risk factor, such as living in a neighbourhood with a poor built environment feature. It is a function of the prevalence of the population exposed to the built environment feature and the impact that exposure to that feature has on physical inactivity [27]. To our knowledge, existing studies have not estimated the PAR% for physical inactivity attributable to different features of the neighbourhood built environment.

The objectives of this study were: (1) to determine the independent associations between different neighbourhood built environment features and physical inactivity within youth, and (2) to estimate the proportion of physical inactivity within the youth population that is attributable to these same built environment features. These objectives were addressed using a national study of Canadian youth in grades 6–10.

## Methods

### Data sources

The relationship between neighbourhood built environment features and physical inactivity was examined within the 2009/10 Canadian Health Behaviour in School-Aged Children Survey (HBSC). The HBSC is made up of two components: (1) a general health survey conducted on a representative sample of 26,078 Canadian youth in grades 6–10 from 436 schools across the country, and (2) GIS measures of the built environment in the neighbourhoods surrounding the participating schools.

The 2009/10 HBSC is a cross-national survey conducted in affiliation with the World Health Organization. This study was limited to the Canadian sample. The HBSC survey covered several aspects of health, health behaviours, and physical and social determinants of health. The Canadian sample was designed according to the international HBSC protocol [28]. The strategy followed a systematic multi-stage cluster technique, whereby individual students are nested in school classes that are nested within schools and school boards. The 2009/10 Canadian HBSC included 26,078 students with distributions reflecting the distribution of Canadians in grades 6–10 (approximate age range 11–15 years) from 436 schools. All provinces and territories in Canada participated with the exception of Prince Edward Island and New Brunswick. Students enrolled in private, special needs, or home schools, as well as incarcerated youth, were excluded; combined they contribute to <10% of the Canadian youth population. Consent was obtained and provided by school boards, individual schools, participants, and their parents/guardians. Ethics approval was obtained from the General Research Ethics Board of Queen’s University.

In this study we used a 1 km radius circular buffer around schools as a proxy for the home neighbourhood. It was not feasible to measure all of the built environment constructs using each student’s home as the centre point of the environment. We therefore attempted to limit the sample of 26,078 students to those students who lived within the 1 km circular buffer of their school. A 1 km distance represents a 10–15 minute walking time and is an appropriate distance for this age group (ie, younger youth do not typically travel >1 km from home unsupervised) [29]. Since many students from the full sample do not live within a 1 km distance of their schools, they were excluded from the analyses. Based on the centre of the geographic area covered by each student’s postal code (which within Canadian cities are quite small and typically cover one or two blocks), we calculated the straight line distance between their house and school. Those students living more than 1 km from their school were excluded. Alternatively, for the 40% of participants who did not indicate their postal code on the survey, distance to school was estimated based upon their reported mode and travel time to school from two survey questions. Students with excessive travel time (i.e., > 15 minutes by walking or >5 minutes by bicycle or motorized transportation) were assumed to live more than 1 km from their school and were therefore excluded (n = 8,917 students). Third, students with missing information on the physical activity outcome (n = 8.669) and/or potential covariates were excluded (n = 8,032). Lastly, schools where all built environment features could not be measured were excluded (n = 6,626). The final sample consisted of 6,626 students from 272 schools.

### Physical inactivity outcome

Information on physical activity behaviours was collected from the average of students’ responses to two questions: *“Over a typical or usual week, on how many days are you physically active for a total of at least 60 minutes per day?”* and *“Over the past 7 days, on how many days were you physically active for a total of at least 60 minutes per day?”* Reliability estimates demonstrate a good level of agreement between the two survey questions (Cronbach’s alpha = 0.79), and the mean of the two items performed better in reliability and validity analyses than either item separately [30]. Validation studies demonstrate a good test-retest reliability for these two questions (67 to 85% agreement; intra-class correlation value of 0.71) and the questionnaire responses are correlated with objective measures of physical activity obtained by accelerometry (r = 0.39) [31,32]. Participants were placed into one of two groups based on their average response to the two questions: physically inactive (≤4 days per week) vs. physically active (> 4 days per week). Although the physical activity guidelines in Canada recommend 60 minutes of moderate-to-vigorous physical activity daily [2], the cut-point of ≤4 days per week was chosen as it corresponded to the lowest ~25% of the sample and simplified interpretation of the data.

### Neighbourhood built environment features

The addresses of the 272 schools were mapped in ArcGIS and a 1 km circular radius buffer was constructed around every school. Several built environment features were measured within these 1 km buffers. Features were selected based on their established associations with physical inactivity and/or obesity in young people and their availability to the research team through national GIS data [8,33]. As explained in more detail below, we grouped together built environment features that measured a similar construct to investigate the following environmental influences: walkability, outdoor play areas, recreation facilities, and aesthetics.

#### Walkability

Six walkability items were measured: intersection density [10], average block length [10], connected node ratio [10], mixed land use [11,12], road speeds [13], and sidewalk coverage [34,35]. Measures were obtained using ArcGIS software with CanMap® Streetfiles, CanMap® Route Logistics, and Google Earth Streetview imaging. Intersection density is the number of intersections per unit of area [10], and was calculated by dividing the total number of real nodes by the land area within the neighbourhood buffer. Connected node ratio refers to proportion of intersections within a buffer that are 3 or 4-way intersections [10], and was determined by dividing true street intersections by the total number of intersections, including cul-de-sacs and dead ends. Average block length was calculated by dividing the total length of roads in each buffer by the number of true intersections [10]. Land-use mix refers to the types of buildings that comprise an area [11], and for this study consisted of the percentage of total land area comprised of residential area [20,26]. Road speed was measured as the percentage of the total road distance within the buffer that was comprised of low speed roads, defined here as having a speed limit ≤50 km/h. Sidewalk coverage was measured as the percentage of the total road distance within the buffer that was comprised of roads with a sidewalk on one or both sides [36].

We created a summary walkability scale based upon the six items using principal component analysis. Results revealed one factor that accounted for 60% of the variance. The four variables that loaded onto this factor and their factor loadings were: land-use mix (0.84), road speeds (0.80), intersection density (0.75), and sidewalk coverage (0.71). The Anderson-Rubin method was used to calculate a summary walkability z-score for the components that were derived from the principal components analyses. These summary scores were subsequently divided into quartiles.

#### Outdoor play spaces

Four outdoor play space items were measured: parks and other public green spaces [15,16,22], open wooded areas, the presence of cul-de-sacs on neighbourhood roads [17-19,37], and presence of yards at home [18,37]. Measures were obtained using CanMap® Streetfiles, CanMap ® Route Logistics*,* CanMap® Parks and Recreation, and Google Earth Streetview Imaging in ArcGIS. The proportion of total land area devoted to parks and other public green spaces (including national parks, provincial parks, territorial parks, and municipal parks/sportsfields) was calculated for each buffer [22,38], and the neighbourhoods were divided into quartiles. The same was done for wooded areas, however, since 40% of students lived in neighbourhoods with no wooded areas, a ‘none’ category was created and tertiles were created for the remaining 60% of the neighbourhoods. Cul-de-sac density was measured by subtracting the number of true intersections from the total number of intersections in each school buffer and dividing by the land area of the buffer [39]. To measure the presence of yards at home, 15 observations points were plotted in each 1 km buffer in an evenly spaced grid (approximately 500 m apart in the X and Y directions from the buffer’s centre). Within Google Earth Streetview, a 360 degree panoramic view was taken at each of the 15 points to measure the proportion of houses and other buildings that had a yard in front using the following scale: 0 = *‘none’,* 1 = *‘less than ¼’,* 2 = *‘¼ to ½’,* 3 = *‘more than half to ¾’* and 4 *= ‘more than ¾ to all’.* A summary yard score was created for each buffer by summing the scores from all 15 points, so that each buffer had a score ranging from 0–60. A principal component analysis investigating the outdoor play space measures revealed little agreement between them (Cronbach’s alpha < .10). Each measure was therefore examined as an individual exposure variable.

#### Recreation facilities

The number of recreation facilities was measured within each buffer using ArcGIS software and the Enhanced Points of Interest database (DMTI Spatial Inc., 2009). Standard Industrial Classification codes were identified and summed within each buffer for the following facilities: dance studios and halls, bowling centers, physical fitness facilities, public golf courses, membership sports and recreation clubs, and amusement and recreation not elsewhere classified. The total number of recreation facilities within each buffer was summed and divided by the buffer land area to develop a measure of recreation facility density.

#### Aesthetics

Three aesthetics items were measured: amount of litter, amount of graffiti, and overall visual condition of buildings and grounds. Measures were obtained using CanMaps Streetfiles® and Google Earth Streetview Imaging. As explained in detail elsewhere, criteria used to assess the items were based on physical disorder studies and measurements were obtained using a 360 degree panoramic view and subjective ratings of 15 points within each buffer [40]. The measure of litter was based on a scale that ranged from *0 = ‘a considerable amount (more than 20 pieces)’ to 4 = ‘none (no litter).*’ Amount of graffiti measured was based on a scale from *0 =* ‘*a considerable amount (more than 5 tags)’* to *3 = ‘none (no graffiti).’* The condition of buildings and grounds was scored from *0 = ‘poor (major overhaul needed to improve appearance of buildings)’* to *3 = ‘excellent (most buildings in immaculate condition).’* Since 15 points were investigated within each buffer, summary scores ranged from 0–60 for litter and from 0–45 for graffiti and the conditions of buildings and grounds. The intra-rater and inter-rater reliability coefficients for repeated Google Earth street view assessments ranged from 0.78 to 0.99 and from 0.65 to 0.99, respectively, for these items [40]. Google Earth street view assessments are well correlated to scores obtained by in-person assessments, with correlation values ranging from 0.65 to 0.99 [40].

A principal component factor analysis based on the three measures was used to create a summary aesthetic score. There was agreement between the three variables, and together they accounted for 54% of the variance (Cronbach’s alpha = 0.63). Factor loadings were 0.81 for litter, 0.75 for graffiti, and 0.65 for conditions of buildings and grounds. The Anderson-Rubin method was used to calculate a summary aesthetics z-score which was subsequently divided into quartiles.

### Covariates

Covariates were chosen based on their demonstrated associations with physical activity, their inclusion in prior research on the built environment and physical activity in young people, and their availability within the HBSC database [6,7,41,42]. Variables considered as potential covariates at the individual-level consisted of gender, age, race (Caucasian, other), and family socioeconomic status (SES). Family-SES was obtained using a measure of perceived family wealth based on responses to the question: “*How well off do you think your family is?*” Participants were categorized into low (*“not very well off”* or *“not at all well off”)*, low-medium (*“average”)*, medium-high (*“quite well off”)* and high (*“very well off”*) groups [43].

Neighbourhood level covariates included neighbourhood-SES, population density, and climate. Neighbourhood-level SES and population density were captured from the 2006 Canadian Census data in PCensus for Mappoint (Tetrad Computer Applications Inc., Vancouver, BC) software was used in combination with ArcGIS software. To determine neighbourhood-level SES, three census measures were considered within each buffer: average family income, unemployment rate, and education (% of adults with less than high school education) [44]. Principal component analysis indicated good agreement between the three variables (Cronbach’s alpha = 0.76) with factor loadings of 0.87 (income), 0.75 (unemployment), and 0.84 (education). A summary neighbourhood-SES score was created using the Anderson-Rubin method and was divided into quartiles. Population density was calculated by dividing the population in each census block by the land area. Average temperature (°C) and average annual precipitation (cm) for the two month period prior to survey administration were obtained from the closest weather station to each neighbourhood buffer using the Environment Canada national climate archive [45].

### Statistical analyses

All analyses were performed in SAS version 9.2 (SAS Inc., Cary, NC). Conventional descriptive statistics were used to describe the study sample and neighbourhood-level characteristics. Multilevel logistic regression analysis was used to examine the relationship between the neighbourhood built environment features and physical inactivity. All categorical variables were entered into the models such that the referent group was the most optimal category for physical activity. This allowed us to present all of the associations as risk factors rather than as a combination of risk and protective factors. Prior to the model building process, an empty model was run to calculate an intra-class correlation (ICC), which indicated the proportion of variance in physical inactivity explained by neighbourhood-level differences [46]. An ICC value of 9% was found, justifying the use of multi-level modeling.

For the model building process, we initially ran bivariate multilevel logistic regression models for each built environment feature and each covariate. This was followed by development of a series of multivariate models that were created with a systematic approach. First, all individual-level variables were entered into the model (multivariate model 1). Backwards elimination determined the individual-level variables to retain for subsequent models, based on a significance level of p < 0.05. Next, neighbourhood-level variables were added to the reduced individual-level model to create multivariate model 2. Backwards selection methods were then performed for the area-level variables based on a significance level of p < 0.05 to derive the multivariate model 3, which was the final model. Variables found to be significant at p < 0.05 based on the p-trend, the p-value for any individual category, or the average p-value for all categories were retained in the model building process.

All of the multilevel logistic regression models were fit as generalized linear models using the SAS GLIMMIX procedure, with a binomial distribution and a logit link. This accounted for both the clustered and hierarchical nature of the data. To optimize convergence of the multilevel models, a Newton–Raphson with ridging technique was applied [47].

Since physical inactivity is not a rare outcome, the odds ratios (OR) obtained from the logistic regression analyses do not approximate relative risks (RR) [48]. The ORs were therefore transformed to RRs using the following formula: RR = OR / [(1 – P) + (OR × P)] where P represents the prevalence of physical inactivity in each exposure group for the built environment variables [48].

PAR% estimates were calculated to determine the proportion of physical inactivity attributable to features of the built environment that were retained in the final model. These were based upon the RR values produced in the final model and the prevalence of the sample in the relevant neighbourhood built environment exposure groups. The equation is: PAR% = [P(RR – 1)/1 + P (RR – 1)]. For variables with more than two exposure categories with a significantly increased RR, individual PAR% calculations for the non-referent categories were summed to create an overall PAR% value.

## Results

Individual characteristics of the 6,626 participants are presented in Table 1. The mean age was 13.4 years with an even distribution of males and females. The majority of the sample was Caucasian (73%) and 10% were of low SES. Table 2 describes the features of the 272 neighbourhoods. The provincial/territorial representation of schools was as follows: Alberta (36), British Columbia (37), Manitoba (12), Newfoundland and Labrador (13), Nova Scotia (4), Nunavut and Northwest Territories (9), Ontario (53), Quebec (41), Saskatchewan (53), and Yukon (14).

**Table 1:** Individual-level characteristics of study sample (n = 6,626)

**Variable**	**N (%)**
**Gender**	
Male	3296 (49.7)
Female	3330 (50.3)
**Age**	
≤11 years	1134 (17.1)
12 years	1524 (23.0)
13 years	1428 (21.6)
14 years	1199 (18.1)
≥15 years	1341 (20.2)
**Race**	
Caucasian	4857 (73.3)
Other	1769 (26.7)
**Family socioeconomic status**	
High	1531 (23.6)
Medium-high	2105 (31.8)
Low-medium	2304 (34.8)
Low	656 (9.9)
**Days per typical week physically active ≥ 60 min**	
0	194 (2.9)
1	353 (5.3)
2	619 (9.3)
3	964 (14.6)
4	1036 (15.6)
5	1286 (19.4)
6	1022 (15.4)
7	1152 (17.4)

**Table 2:** Neighbourhood-level characteristics (n = 272 neighbourhoods)

**Variable**	**Median (interquartile range)**
**Walkability measures**	
Intersection density (number per km^2^)	44.9 (30.2-54.1)
Average block length (km)	0.24 (0.22-0.27)
% of intersections that are 3- or 4-way	84.7 (78.2-90.8)
% roads that are low speed (≤50 km/h)	89.4 (71.0-97.1)
% roads covered by sidewalks	54.7 (40.9-78.6)
Mixed land use (% residential or commercial)	63.2 (45.6-75.0)
**Outdoor play area measures**	
Parks space (% land area)	2.13 (0.06-6.20)
Wooded areas (% land area)	1.76 (0.00-10.5)
Yards at home (scale of 0–60)	42 (37–44)
Density of cul-de-sacs (number per km^2^)	6.4 (3.8-11.8)
**Recreation facilities (number per km**^**2**^**)**	1.3 (0.71-2.23)
**Aesthetics measures**	
Condition of buildings and grounds (scale of 0–45)	29 (26–31)
Graffiti (scale of 0–45)	0 (0–1)
Presence of litter (scale of 0–60)	17 (12–21)
**Socioeconomic status measures**	
Education (% no certificate, diploma or degree)	22.8 (18.0-27.6)
Average employment income ($ CAD)	32,454 (28,374-38,185)
Unemployment rate (%)	4.5 (3.2-6.6)
**Climate measures**	
Average temperature (°C)	−2.3 (−9.4-5.3)
Average precipitation (cm per year)	52.8 (19.2-78.4)
**Population density (per km**^**2**^**)**	12,635 (3,801-64,440)

Table 3 summarizes the bivariate and adjusted (multivariate model 1) associations between the individual-level covariates and physical inactivity. All individual-level covariates were independently associated with physical inactivity, and were therefore retained for subsequent models. The results suggested that females (RR 1.25, 95% CI 1.16-1.33), non-Caucasians (RR 1.30, 95% CI 1.20-1.40), individuals in the lowest SES quartile (RR 1.38, 95% CI 1.22-1.54), and the oldest students (RR 1.25, 95% CI 1.09-1.41) were more likely to be physically inactive.

**Table 3:** Bivariate and multivariate (Model 1) relationships between individual-level characteristics and physical inactivity in Canadian youth (n = 6,626)

	**% Physically inactive**	**Bivariate model**	Multivariate model 1*
		**RR (95% CI)**	**RR (95% CI)**
**Gender**			
Male	23.5	1.00	1.00
Female	30.5	1.26 (1.17-1.34)	1.25 (1.16-1.33)
*P value*	<.0001	<.0001	<.0001
**Age**			
≤11	27.7	1.00	1.00
12	24.0	0.91 (0.78-1.04)	0.91 (0.79-1.05)
13	25.4	1.07 (0.92-1.21)	1.08 (0.94-1.24)
14	26.9	1.01 (0.87-1.18)	1.04 (0.89-1.21)
**≥**15	31.7	1.24 (1.08-1.40)	1.25 (1.09-1.41)
*P trend*	.002	.0007	.0005
**Race**			
Caucasian	24.5	1.00	1.00
Other	33.8	1.29 (1.19-1.39)	1.30 (1.20-1.40)
*P value*	<.0001	<.0001	<.0001
**Family SES**			
Highest	23.5	1.00	1.00
Medium-high	25.1	1.09 (0.97-1.21)	1.08 (0.96-1.21)
Low-medium	29.7	1.27 (1.17-1.39)	1.26 (1.14-1.38)
Lowest	32.0	1.39 (1.24-1.55)	1.38 (1.22-1.54)
*P trend*	<.0001	<.0001	<.0001

RR (95% CI) = relative risk (95% confidence interval).

* RR estimates for each individual-level variable in multivariate model 1 are adjusted for the individual-level variables shown in the table.

**Figure 1:**
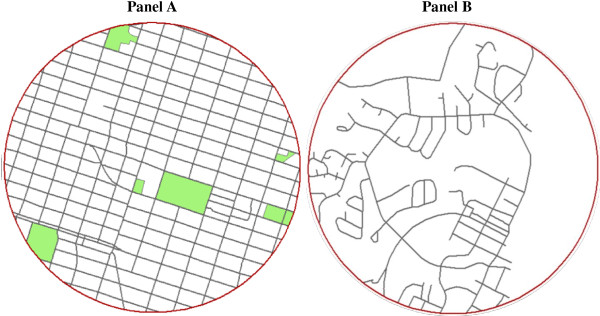
**A 1 km circular radius buffer around two of the schools included in this study.** Park space is represented by the green areas and the street network is shown with the grey lines. **Panel A** is an example of a least ideal neighbourhood (e.g., highly walkable, low density of cul-de-sacs, moderate park space) and **Panel B** is an example of a most ideal neighbourhood (e.g., low walkability, high density of cul-de-sacs and dead ends, low park space) for physical activity in youth.

Table 4 displays the associations between each neighbourhood built environment feature and physical inactivity prior to (bivariate) and after (multivariate model 2) adjusting for individual-level covariates. In the adjusted model, the neighbourhood walkability score, density of cul-de-sacs in the neighbourhood, and proportion of neighbourhood land area devoted to park space were all associated with physical inactivity (P trend < 0.05). These associations remained significant after further adjustment for relevant neighbourhood level covariates (temperature and precipitation), as presented in the final multivariate model in Table 5. The results of the final model indicated that, by comparison to youth living in the least walkable neighbourhoods, the risks for physical inactivity were 28-44% higher for youth living in neighbourhoods in the remaining three walkability quartiles. By comparison to youth living in neighbourhoods with the highest density of cul-de-sacs, risks for physical inactivity were 28-32% higher for youth living in neighbourhoods in the lowest two quartiles. Finally, by comparison to youth living in neighbourhoods with the least amount of park space, risks for physical inactivity were 28-37% higher for youth living in the neighbourhoods with a moderate to high (group 2 and 3) amount of park space. Figure 1 illustrates an example of a least ideal neighbourhood (e.g., highly walkable, low density of cul-de-sacs, moderate to high park space) and most ideal neighbourhood (e.g., low walkability, high density of cul-de-sacs and dead ends, low park space) based on the associations presented in Table 5.

**Table 4:** Bivariate and multivariate (Model 2) relationships between neighbourhood-level characteristics and physical inactivity in Canadian youth (N = 6,626)

	**% Exposed**	**% Physically inactive**	**Bivariate model**	**Multivariate model 2**
			**RR (95% CI)**	RR (95% CI) *
**Walkability score**				
1 (least walkable)	25.4	23.6	1.00	1.00
2	24.4	26.6	1.32 (1.09-1.57)	1.28 (1.06-1.52)
3	25.1	29.0	1.27 (1.06-1.48)	1.21 (1.01-1.42)
4 (most walkable)	25.1	28.8	1.41 (1.18-1.64)	1.32 (1.10-1.56)
*P trend*		.0002	.001	.011
**Outdoor play areas**				
***Yards at home***				
4 (most)	32.2	25.5	1.00	1.00
3	18.0	24.1	0.87 (0.69-1.09)	0.86 (0.69-1.09)
2	23.9	28.3	1.16 (0.97-1.37)	1.17 (0.98-1.39)
1 (least)	26.0	29.6	1.17 (0.97-1.39)	1.13 (0.92-1.36)
*P trend*		.001	.024	.066
***Density of cul-de-sacs***				
4 (most)	25.2	24.9	1.00	1.00
3	23.7	26.6	1.15 (0.93-1.39)	1.17 (0.96-1.40)
2	25.1	29.0	1.24 (1.01-1.48)	1.23 (1.01-1.47)
1 (least)	26.1	27.4	1.25 (1.01-1.51)	1.27 (1.04-1.52)
*P trend*		.039	.026	.019
***Park space***				
1 (least)	25.0	21.6	1.00	1.00
2	24.9	28.0	1.45 (1.19-1.71)	1.42 (1.17-1.68)
3	25.2	29.0	1.50 (1.27-1.75)	1.43 (1.20-1.67)
4 (most)	24.9	29.4	1.43 (1.19-1.69)	1.33 (1.09-1.58)
*P trend*		<.0001	.002	.025
***Wooded areas***				
1 (most)	20.0	25.8	1.00	1.00
2	20.0	26.5	1.07 (0.83-1.35)	1.06 (0.83-1.34)
3	20.0	29.4	1.19 (0.96-1.45)	1.17 (0.94-1.42)
4 (none)	40.1	26.7	1.18 (0.96-1.43)	1.11 (0.89-1.35)
*P trend*		.929	.094	.365
**Recreation facility density**				
4 (most)	25.0	26.0	1.00	1.00
3	26.4	26.4	1.17 (0.96-1.41)	1.17 (0.96-1.41)
2	25.6	29.0	1.02 (0.83-1.24)	1.08 (0.88-1.29)
1 (least)	23.1	27.4	0.95 (0.75-1.16)	0.99 (0.81-1.21)
*P trend*		.078	.322	.724
**Aesthetics**				
1 (best)	24.8	24.5	1.00	1.00
2	24.7	25.7	0.96 (0.78-1.18)	0.96 (0.78-1.18)
3	25.0	25.6	0.94 (0.76-1.15)	0.88 (0.70-1.07)
4 (worst)	25.6	32.1	1.28 (1.08-1.49)	1.16 (0.97-1.36)
*P trend*		<.0001	.006	.158
**Socioeconomic status**				
1 (highest)	24.7	23.5	1.00	1.00
2	25.2	27.7	1.16 (0.95-1.41)	1.10 (0.89-1.34)
3	24.8	28.1	1.18 (0.96-1.41)	1.08 (0.88-1.30)
4 (lowest)	25.2	28.6	1.15 (0.95-1.39)	1.12 (0.92-1.34)
*P trend*		.0014	.171	.158
**Average temperature**				
4 (highest)	24.2	26.9	1.00	1.00
3	27.3	24.3	0.86 (0.69-1.05)	0.82 (0.67-1.00)
2	23.8	28.7	1.02 (0.83-1.23)	1.06 (0.88-1.26)
1 (lowest)	24.7	28.4	1.09 (0.88-1.32)	1.14 (0.93-1.37)
*P trend*		.060	.164	.081
**Average precipitation**				
1 (least)	22.9	27.7	1.00	1.00
2	26.3	23.1	0.96 (0.73-1.25)	0.97 (0.73-1.26)
3	23.3	29.9	1.13 (0.91-1.38)	1.13 (0.91-1.38)
4 (most)	27.4	27.7	1.09 (0.85-1.36)	1.13 (0.89-1.40)
*P trend*		.150	.213	.118
**Population density**				
1 (lowest)	24.9	23.9	1.00	1.00
2	25.1	26.4	1.12 (0.86-1.41)	1.14 (0.89-1.43)
3	24.7	28.0	1.18 (0.96-1.42)	1.16 (0.95-1.39)
4 (highest)	25.3	29.6	1.26 (1.04-1.50)	1.14 (0.94-1.37)
*P trend*		<.0001	.017	.197

RR (95% CI) = relative risk (95% confidence interval).

* RR estimates for neighbourhood-level variables in multivariate model 2 are adjusted for individual-level covariates (gender, age, race, family socioeconomic status).

**Table 5:** Final multivariate model of the relationship between individual- and area-level characteristics with physical inactivity in Canadian youth (n = 6,626)

**Characteristics**	Multivariate model 3*
	**RR (95% CI)**
***Individual-level characteristics***	
**Gender**	
Male	1.00
Female	1.17 (1.12-1.23)
*P value*	<.0001
**Age**	
≤11	1.00
12	0.90 (0.79-1.05)
13	1.09 (0.94-1.26)
14	1.06 (0.88-1.25)
≥15	1.32 (1.12-1.54)
*P trend*	.0003
**Race**	
Caucasian	1.00
Other	1.32 (1.20-1.45)
*P value*	<.0001
**Family SES**	
Highest	1.00
Medium-high	1.08 (0.96-1.21)
Low-medium	1.26 (1.15-1.38)
Lowest	1.38 (1.23-1.53)
*P trend*	<.0001
***Neighbourhood-level characteristics***	
**Walkability score**	
1 (least walkable)	1.00
2	1.28 (1.06-1.54)
3	1.29 (1.06-1.57)
4 (most walkable)	1.44 (1.18-1.74)
*P trend*	.002
**Density of cul-de-sacs**	
4 (most)	1.00
3	1.08 (0.87-1.33)
2	1.28 (1.04-1.55)
1 (least)	1.32 (1.07-1.60)
*P trend*	.001
**Park space**	
1 (least)	1.00
2	1.37 (1.10-1.65)
3	1.28 (1.02-1.56)
4 (most)	1.14 (0.90-1.42)
*P trend*	.378
**Average temperature**	
4 (highest)	1.00
3	0.86 (0.72-1.04)
2	1.17 (0.96-1.41)
1 (lowest)	1.32 (1.08-1.59)
*P trend*	.015
**Average precipitation**	
1 (least)	1.00
2	1.11 (0.85-1.40)
3	1.23 (0.97-1.54)
4 (most)	1.32 (1.04-1.63)
*P trend*	.009

RR (95% CI) = relative risk (95% confidence interval).

*RR estimates for each variable are adjusted for all other variables listed in the table.

PAR% estimates for physical inactivity for the neighbourhood built environment features that were retained in the final multivariate model are displayed in Table 6. PAR% estimates suggested that 23.3% of physical inactivity was attributable to living in walkable neighbourhoods, 16.2% was attributable to living in neighbourhoods with a low density of cul-de-sacs, and 15.0% was explained by living in neighbourhoods with a moderate to high amount of park space.

**Table 6:** Population attributable risk (PAR%) for physical inactivity among Canadian youth for relevant built environment features (n = 6,626)

**Built environment feature**	**% Exposed**	**Population**
		**Attributable risk%**
**Walkability score**		
1 (least walkable)	24.7	**--**
2	25.3	6.6
3	24.5	6.6
4 (most walkable)	25.5	10.1
**Density of cul-de-sacs**		
4 (most)	25.2	**--**
3	23.7	1.9
2	25.1	6.6
1 (least)	26.1	7.7
**Park space**		
1 (least)	25.0	--
2	24.9	8.4
3	25.2	6.6
4 (most)	24.9	--

## Discussion

This national Canadian study examined the associations between several features of the neighbourhood built environment and physical inactivity in youth and estimated the proportion of physical inactivity within the youth population that is attributable to neighbourhood built environment features. Neighbourhood walkability, density of cul-de-sacs, and park space were independently related to physical inactivity, although these associations were modest in strength. Nonetheless, because the prevalence of youth residing in non-ideal neighbourhoods was high, a high proportion of physical inactivity within the population was attributable to these three neighbourhood built environment features.

A key difference in the current study and previous studies examining the associations between the neighbourhood built environment and physical activity within youth is that our study considered multiple built environment features and involved a geographically diverse sample from across the country. Previous studies did not simultaneously examine all relevant built environment features and typically studied small samples from small geographic regions (e.g., a single city).

The relationship between walkability and total physical inactivity observed in this study and some other studies of youth [49-51] is opposite to what has been shown in adults [52,53]. Adults from neighbourhoods with greater walkability have higher total physical activity levels than adults from neighbourhoods with a lower walkability [53], while youth from neighbourhoods with greater walkability have lower total physical activity levels than youth from neighbourhoods with a lower walkability [49-51]. The opposite patterns observed for adults and youth is likely explained by the different forms of activity that they tend to engage in. While active transportation is the most common method in which adults engage in physical activity [54], it only accounts for a small proportion of youths’ total physical activity [55,56]. A much greater proportion of youths’ total physical activity is made up of active play and organized sport [57]. As such, the negative relationship between neighbourhood walkability and total physical activity in youth reported here and in other studies may reflect that features of highly walkable neighbourhoods inhibit active play and/or sport. There may be greater traffic and safety concerns in highly walkable urban neighbourhoods where the streets and houses are tightly packed together (left panel of Figure 1) which may act as a barrier for youth to go outside and engage in sport and play as they might do in less busy and populated areas [13]. Indeed, in our study sample perceptions around heavy traffic and it being unsafe in the neighbourhood for young children to play outdoors were slightly more prominent in the most walkable neighbourhoods (data not shown).

Although neighbourhood parks and public green spaces provide a freely accessible space for youth to be active, such space was not independently associated with physical inactivity in the present study. Thus, while park space appears to influence the physical activity and body weight of younger children (e.g., <12 years old) [15,38], the results from this and other studies [20,58,59] suggest that this is not the case in older children and adolescents. Indeed, park users are primarily younger children and older adults [38,60], and the amenities in most neighbourhood parks (e.g., monkey bars, slides, swings, etc.) are better suited to younger children than to adolescents [59]. Adolescents may travel outside of their home neighbourhood to use community parks with courts and fields, as these amenities are more suitable for the types of activities they engage in [61].

Many youth engage in physical activity in public spaces designed for motorized vehicles such as streets and parking lots [37]. Our findings suggest that the risk of physical inactivity is increased by 30% for youth residing in a neighbourhood with a low density of cul-de-sacs. Similar associations have been found in the US and Australia [20,56]. Cul-de-sacs may encourage physical activity by providing an open area for youth to participate in unorganized sport and play (e.g., street hockey, catch, skateboarding) in close proximity to their home.

Consistent with our findings, a recent literature review concluded that the number/density of neighbourhood recreational facilities, as measured objectively using GIS, is not associated with physical activity in youth [9]. Aspects such as fees, quality, and accessibility of these facilities may be more relevant for adolescent use than presence alone [21]. Furthermore, recreational facilities may be more important at the community level than at the neighbourhood level. That is, youth are often driven outside of their neighbourhood to participate in organized team and club sports occurring at facilities in other areas of their extended community [62].

Previous studies examining the relationship between neighbourhood aesthetics and physical activity in youth have reported positive [25,26,63], negative [64], and null [65,66] associations. Thus, as suggested by our findings, there does not appear to be a clear and consistent effect of aesthetics on physical activity within youth. Youth living in aesthetically unpleasant neighbourhoods may become immune to its aesthetic features, and such features of the environment may not be responsible for deterring physical activity [8].

The PAR% values for physical inactivity for the three neighbourhood built environment features independently associated with physical inactivity were 23% for moderate or high walkability, 16% for low cul-de-sac density, and 15% for a moderate amount of park space. This suggests that the neighbourhood built environment has a meaningful impact on youths’ physical inactivity at the population level. This also suggests that these three built environment features would be key targets if the only goal was to improve youths’ physical activity levels. However, as discussed above, these built environment features appear to impact physical activity differently in adults, youth, and children. Thus, it will be challenging to optimize the built environment for the entire population. For example, optimizing street connectivity and walkability to increase physical activity within adults may have an adverse impact on youths’ total physical activity levels. An alternative that may suit all ages would be to design neighbourhoods with poorly connected street networks and lots of cul-de-sacs, but with well-connected walking and cycling pathways integrated into the design to facilitate active transportation [67]. Future built environment research that simultaneously studies adults, youth, and children is needed.

Key strengths of this study are the use of a large sample of Canadian youth, the simultaneous consideration of multiple built environment features, and the generalizability of the study methodology. While the findings may only be relevant for Canadian youth living in close proximity to their schools, the use of contemporary statistical modeling, the population attributable risk, and the simultaneous investigation of multiple built environment features is novel.

There are several limitations of this study. The use of a questionnaire to assess physical activity may have led to misclassification of this behaviour as youth tend to misreport activity levels [68,69]. It is likely that this measurement error was non-differential and would have led to underestimated RR and PAR% estimates. Secondly, GIS databases are not always up-to-date, which would have further contributed to non-differential misclassification. Third, this was a cross-sectional study, and therefore we cannot be certain that the observed relations were causal in nature. However, given that youth have limited autonomy in determining where they live, this study was likely not susceptible to reverse causality. Fourth, we did not assess the presence of backyards and driveways at the home, and therefore we may not have fully captured all aspects of the neighbourhood built environment that may influence youths’ physical activity. Fifth, participants were assigned to school neighbourhoods based on the place where they reported that they lived most often; we were not able to account for the fact that some youth may split their time in different homes. Finally, the 2010 Canadian HBSC survey was primarily completed in the colder months of the year when physical activity levels are at their lowest [70], and this may have impacted the associations that were observed.

## Conclusion

The neighbourhood built environment features most strongly associated with physical inactivity in this national study of Canadian youth were high walkability, a low density of cul-de-sacs, and a moderate amount of park space. Some of these associations were in the opposite direction to what has previously been reported in adults and younger children. While the relative risk for physical inactivity associated with exposure to any given built environment feature was low, the prevalence of youth exposed to non-ideal environments was high. Thus, at the population-level, a large proportion of inactivity was explained by the neighbourhood built environment.

The methods and approaches used in this paper have relevance for and could be replicated in other countries. To our knowledge, no previous studies have investigated the cumulative and collective effect of several features of the built environment on youth physical inactivity. Determination of PAR% is also novel and makes a unique contribution to the built environment literature. Since features of the built environment may impact youth physical inactivity differently in different parts of the world, understanding the importance of each individual feature while controlling for the potential confounding from other features of the built environment is important for informing land use planning and zoning policies to improve physical activity opportunities for youth.

## Competing interests

The authors declare that they have not competing interests.

## Authors’ contributions

Authors’ contributions to this paper are as follows: IJ and RL designed research and contributed to the conception of the study. RL assisted in GIS data collection, performed data analysis, and drafted the initial version of the manuscript. IJ was a co-investigator of the Canadian HBSC study, provided advice and input on the analyses, and critically reviewed and edited the manuscript for intellectual content. Both authors have responsibility for the final content and approve the final manuscript.
